# Recurrent Ventricular Fibrillation Induced by a Mobile Thrombus in the Sinus of Valsalva in a Patient With Bipolar Disorder

**DOI:** 10.7759/cureus.111391

**Published:** 2026-06-23

**Authors:** Kensuke Kobayashi, Yusuke Mizuno, Hiroaki Yusa

**Affiliations:** 1 Cardiovascular Surgery, Daiyukai General Hospital, Ichinomiya, JPN; 2 Cardiovascular Medicine, Umejima Heart Clinic, Tokyo, JPN

**Keywords:** bipolar disorder, inflammation, sinus of valsalva, thrombosis, ventricular fibrillation

## Abstract

Mobile thrombi of the sinus of Valsalva are rare and may result in life-threatening complications. We report a surgical case of recurrent ventricular fibrillation caused by a mobile thrombus in the sinus of Valsalva in a patient with bipolar disorder.

A 42-year-old man with bipolar disorder collapsed due to loss of consciousness. Ventricular fibrillation was documented, and return of spontaneous circulation was achieved after cardiopulmonary resuscitation. Recurrent ventricular fibrillation required eight defibrillations. Emergency coronary angiography revealed no significant stenosis, whereas left ventriculography disclosed a mobile intraluminal filling defect in the ascending aorta. Contrast-enhanced computed tomography and echocardiography suggested aortic dissection. Emergency surgery removed an 8×25-mm mobile mass attached 5 mm distal to the left coronary ostium. Histopathology examination confirmed thrombus. Transient obstruction of the left coronary ostium by the mobile thrombus was considered the cause of the recurrent ventricular fibrillation. Bipolar disorder-associated systemic inflammation might have been involved in thrombus formation. Although rare, this entity should be considered in patients with otherwise unexplained malignant ventricular arrhythmias. Early surgical removal followed by appropriate postoperative anticoagulation may help prevent recurrence and improve outcomes.

## Introduction

Ascending aortic thrombus formation is a rare phenomenon because the high-flow hemodynamic environment and elevated wall shear stress within the ascending aorta generally inhibit thrombus development [1]. Mobile mural thrombi of the ascending aorta, including those involving the aortic root and aortic arch, are associated with a high risk of catastrophic thromboembolic events such as myocardial infarction, ischemic stroke, and mesenteric ischemia [2,3]. However, this condition remains poorly characterized because the available evidence is limited to isolated case reports and small case series, and no consensus has been established regarding its etiology, diagnostic approach, or optimal management [2,3]. Although cerebral and peripheral embolic events are well-recognized manifestations of mobile aortic thrombi, reports describing coronary malperfusion or myocardial ischemia are relatively uncommon [3]. In particular, the diagnosis of intra-aortic thrombi can be challenging in the emergency setting.

We report a rare case of recurrent ventricular fibrillation caused by a mobile thrombus originating from the left sinus of Valsalva, presumably through transient obstruction of the left coronary ostium, in a patient with bipolar disorder, a mood disorder characterized by recurrent episodes of mania, hypomania, and/or depression as defined in the Diagnostic and Statistical Manual of Mental Disorders, Fifth Edition (DSM-5) [4]. Given the rarity of sinus of Valsalva thrombus and its potential to cause life-threatening ventricular arrhythmias, reporting similar cases may contribute to improved recognition and management of this uncommon condition.

## Case presentation

**Figure 1:**
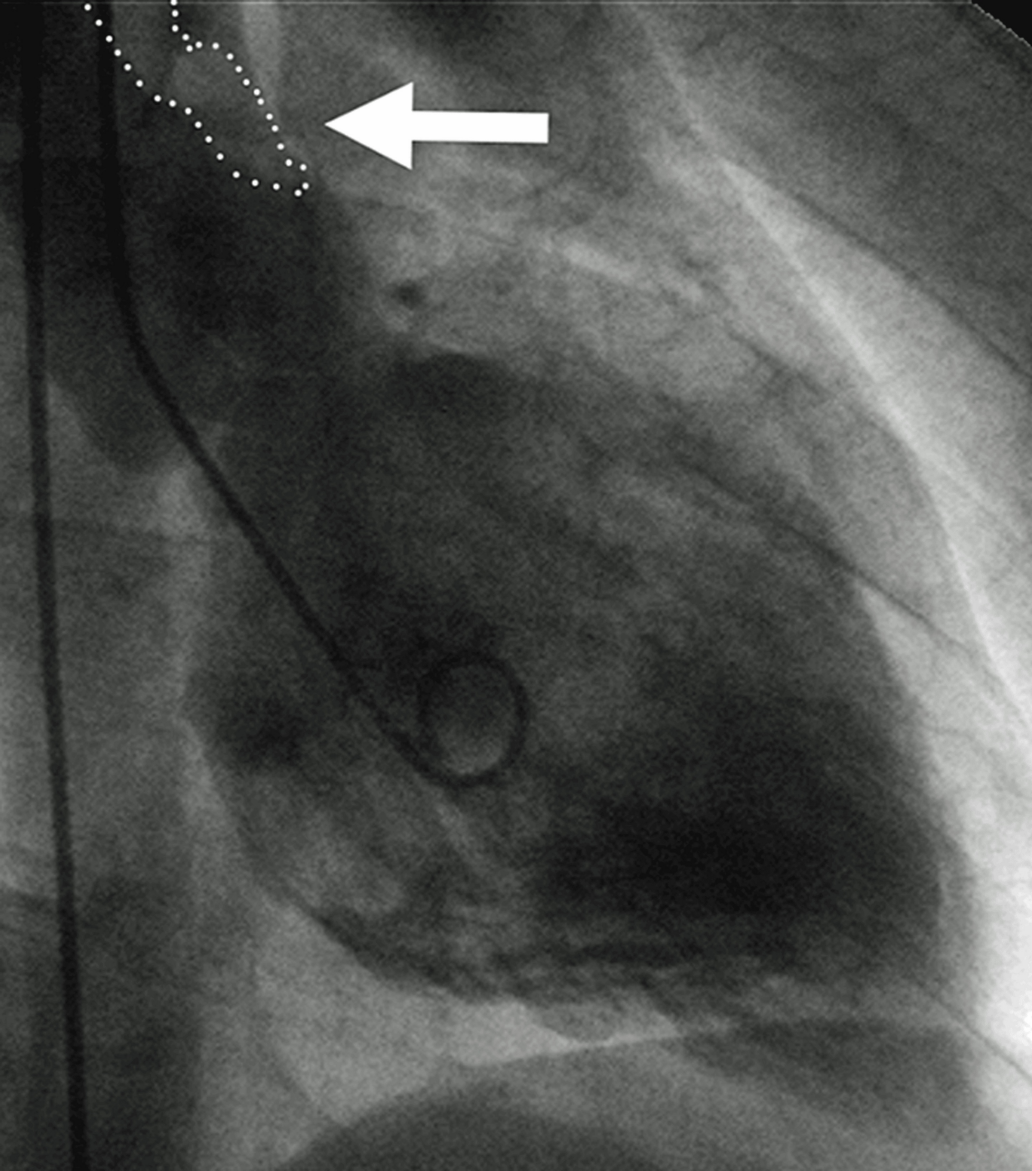
Left ventriculography shows a mobile intraluminal filling defect in the ascending aorta (white arrow).

A 42-year-old man collapsed at home due to loss of consciousness and was transported to our emergency room. He had a history of bipolar disorder, diagnosed according to Diagnostic and Statistical Manual of Mental Disorders, Fifth Edition (DSM-5) criteria, and had been receiving psychiatric treatment with multiple oral medications, including olanzapine. No history of illicit drug use was identified. Ventricular fibrillation was detected in the ambulance, and cardiopulmonary resuscitation was initiated. However, ventricular fibrillation recurred during transport, and after arrival at the emergency room, eight defibrillations were required to restore spontaneous circulation. Initial laboratory investigations revealed marked leukocytosis (white blood cell count: 26,630/μL) and slightly elevated C-reactive protein levels (0.39 mg/dL). Cardiac troponin testing was negative, and the fibrin/fibrinogen degradation product level was mildly elevated at 5.1 μg/mL. Although the electrocardiogram revealed sinus rhythm without ischemic ST-segment changes or T-wave inversion, emergent coronary angiography was performed for suspected acute coronary syndrome, including severe vasospastic angina. The coronary arteries were completely normal; however, left ventriculography revealed a mobile intraluminal filling defect in the ascending aorta (Figure 1). Subsequent contrast-enhanced computed tomography suggested ascending aortic dissection, whereas transthoracic echocardiography demonstrated no abnormality.

**Figure 2:**
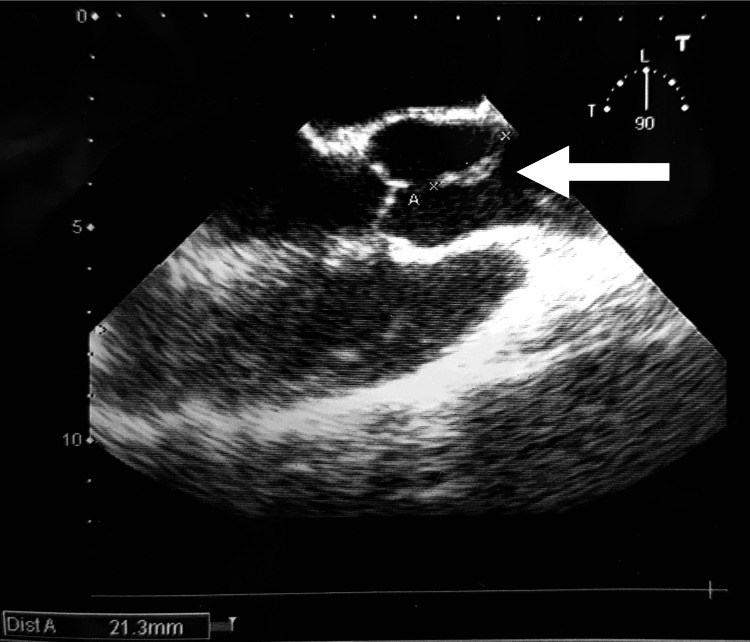
Transesophageal echocardiography shows a highly echogenic, flap-like structure in the ascending aorta (white arrow). Acute aortic dissection was suspected based on these findings.

**Figure 3:**
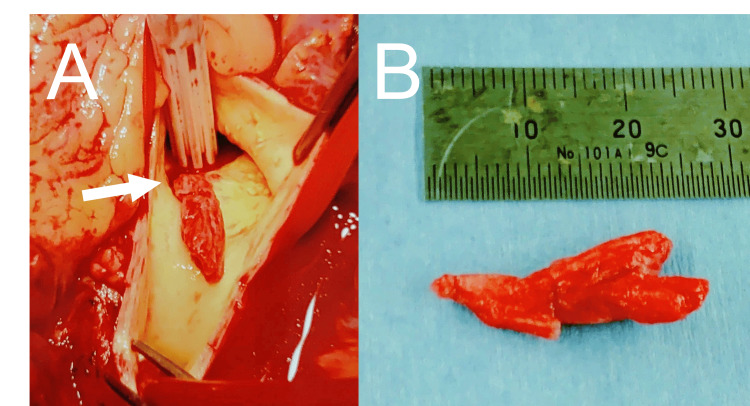
Operative findings show (A) a pedunculated mass arising from the left sinus of Valsalva near the left coronary ostium (white arrow) and (B) the excised mass measuring 8×25 mm.

The patient subsequently underwent an emergency surgery under cardiopulmonary bypass with a plan for ascending aortic replacement. Transesophageal echocardiography revealed a highly echogenic flap-like structure in the ascending aorta (Figure 2). Under deep hypothermic circulatory arrest with antegrade cerebral perfusion, transverse aortotomy revealed neither aortic dissection nor a penetrating atherosclerotic ulcer. The aortic wall was soft and elastic, appearing intact. However, an 8×25-mm mobile mass was detected 5 mm distal to the left coronary ostium, originating from the left sinus of Valsalva. The mass was red and pedunculated, without calcification, and was easily excised using forceps (Figures 3A, 3B). The intimal surface at the attachment site was slightly rough and similar in color to the adjacent intima. Ascending aortic replacement was not performed because no aortic wall pathology was identified.

**Figure 4:**
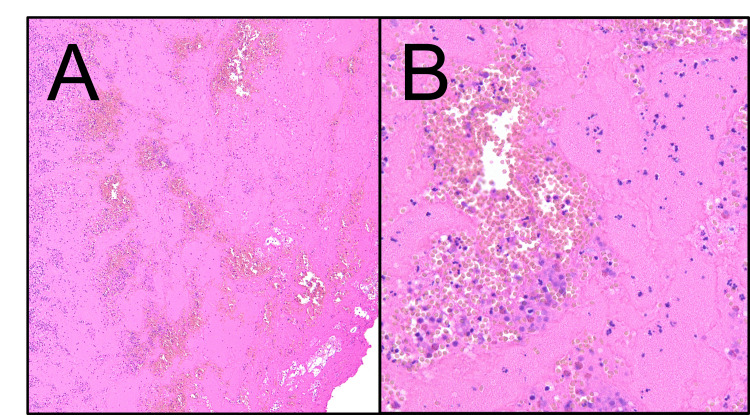
Histopathological findings of the excised mass (hematoxylin and eosin stain). (A) Low-power view showing thrombotic material composed predominantly of eosinophilic fibrin. (B) High-power view demonstrating fibrin-rich thrombus with entrapped erythrocytes. No associated intimal tissue, atheromatous component, or evidence of malignancy was identified.

Histopathological examination confirmed a fibrin-rich thrombus without associated intimal tissue, atheromatous components, or evidence of malignancy (Figures 4A, 4B). The patient had blood type A, Rh-negative. Further laboratory evaluation for hypercoagulability, including protein S activity, protein C activity, lupus anticoagulant, anticardiolipin IgG antibody, and anti-β2-glycoprotein I antibody, revealed no abnormalities. The postoperative course was uneventful, and the patient was discharged on warfarin therapy without neurological deficits or other embolic events. The subsequent outpatient course was unremarkable, with no evidence of recurrent ventricular arrhythmias or thromboembolic complications.

## Discussion

Virchow’s triad - hypercoagulability, hemodynamic changes (stasis or turbulence), and endothelial injury - is considered a fundamental mechanism in the pathogenesis of mobile aortic thrombus [2]. Conditions associated with hypercoagulability include both congenital and acquired factors, such as deficiencies of natural anticoagulants, antiphospholipid syndrome, malignancy, drug-related conditions, and prothrombotic states associated with infection or systemic inflammation.

In the present case, laboratory examination revealed no evidence of hypercoagulability. Potential drug-related factors were considered due to the absence of identifiable coagulation abnormalities. The patient had a long-term history of olanzapine use. Among second-generation antipsychotics, some agents, including olanzapine, have been associated with an increased risk of venous thromboembolism; nevertheless, their association with arterial thrombosis remains unclear [5]. Wang et al. reported a case of acute myocardial infarction associated with clozapine, another second-generation antipsychotic; however, no reports have described an increased risk of arterial thrombosis with olanzapine use [6]. Furthermore, bipolar disorder itself has been suggested to be associated with a prothrombotic state [7]. Fluctuations in mood polarity, including depressive and manic states, may impact endothelial function [7]. Moreover, arterial thrombotic events, including ascending aortic thrombus and acute myocardial infarction, have been reported in patients with bipolar disorder, and the present case adds to the limited evidence demonstrating a possible association between bipolar disorder and arterial thrombosis [3,6].

It is also necessary to consider infection- or inflammation-related mechanisms. Although several cases of ascending aortic floating thrombus have been recently reported in patients with Severe Acute Respiratory Syndrome Coronavirus 2 (SARS-CoV-2) infection, no evidence of concomitant infection was identified in our case [8]. On the other hand, recent studies have suggested that bipolar disorder is associated with chronic low-grade systemic inflammation mediated by pro-inflammatory cytokines and intracellular signaling pathways [9]. Such an inflammatory background may have contributed to thrombus formation in the present case.

Regarding the patient’s blood type, a review of the literature indicates that non-O ABO blood type demonstrates a consistent association with venous thromboembolism; nonetheless, evidence regarding arterial thrombosis remains inconclusive [10]. Conversely, no established association has been reported between other blood group systems, including Rh type, and thrombotic risk.

In the present case, no structural conditions that can induce turbulent flow, such as an aortic aneurysm and aortic stenosis, were identified. Recent research suggests that four-dimensional flow imaging can provide functional and hemodynamic insights into blood flow within the aorta, including vortex flow patterns in the sinus of Valsalva [11]. A detailed evaluation of aortic hemodynamics may help clarify the relationship between flow characteristics and thrombus formation; however, this modality remains investigational and is not feasible in emergency settings.

Endothelial injury is a well-recognized substrate for mural thrombosis, and microerosions of atherosclerotic plaques in the aorta may serve as a nidus for the formation of mobile thrombus [3]. Nevertheless, in the present case, no significant atherosclerotic plaque was identified at the site of attachment.

In previously reported cases of ascending aortic thrombus associated with antiphospholipid syndrome, postoperative medical therapy consisted of combined warfarin and aspirin after thrombectomy [12]. In the present case, warfarin monotherapy was selected because laboratory findings related to hypercoagulability were within normal limits, and the international normalized ratio of prothrombin time (PT-INR) was maintained at 2.0-2.5. However, this strategy has not been established as a standard treatment approach.

Optimal therapeutic strategies range from surgical thrombectomy and/or ascending aortic replacement to conservative anticoagulation, depending on the clinical condition of the patient. Management should be tailored to the individual clinical context [2,3]. Further studies are required to clarify the underlying mechanisms and establish evidence-based treatment strategies for this rare condition.

## Conclusions

Mobile aortic thrombi can occur without identifiable predisposing factors and may result in life-threatening ventricular fibrillation when originating from the sinus of Valsalva. It is essential to recognize this rare but potentially fatal condition in patients presenting with unexplained malignant arrhythmias. Bipolar disorder-associated systemic inflammation may represent a possible contributing factor in thrombus formation, although its pathological significance remains unclear. Early surgical intervention and individualized postoperative anticoagulation may contribute to favorable outcomes.
